# microRNA-34a as a Therapeutic Agent against Human Cancer

**DOI:** 10.3390/jcm4111951

**Published:** 2015-11-16

**Authors:** Yoshimasa Saito, Toshiaki Nakaoka, Hidetsugu Saito

**Affiliations:** Division of Pharmacotherapeutics, Keio University Faculty of Pharmacy, 1-5-30 Shibakoen, Minato-ku, Tokyo 105-8512, Japan; E-Mails: toshiakinakaoka1116@gmail.com (T.N.); saito-hd@pha.keio.ac.jp (H.S.)

**Keywords:** microRNA, miR-34a, cancer, cancer stem cell, DNA methylation

## Abstract

microRNAs (miRNAs) are small non-coding RNAs that down-regulate expression of various target genes. Cancer-related miRNAs are aberrantly expressed and act as tumor suppressors or oncogenes during carcinogenesis. We and other researchers have demonstrated that important tumor suppressor miRNAs are silenced by epigenetic alterations, resulting in the activation of target oncogenes in cancer cells. miR-34a was identified as a target of p53 and induces a G1 cell cycle arrest, senescence and apoptosis in response to DNA damage. miR-34a is an important tumor suppressor whose expression is epigenetically silenced in various human cancers. Enforced expression of miR-34a induces cell cycle arrest, apoptosis, senescence, and suppression of epithelial-mesenchymal transition and inhibits cell proliferation of cancer stem cells. Epigenetic therapy with chromatin-modifying drugs such as inhibitors of DNA methylation and histone deacetylase has shown clinical promise for the treatment of malignancies. Restoring of miR-34a expression by epigenetic therapy and/or delivery of miR-34a mimics may be a promising therapeutic strategy against human cancer.

## 1. Introduction

microRNAs (miRNAs) are 21–25 nucleotides non-coding RNAs that can post-transcriptionally down-regulate the expression of various target genes. Currently, ~2500 human miRNAs have been identified in the human genome, each of which potentially controls hundreds of target genes. miRNAs are expressed in a tissue-specific manner and play important roles in cell proliferation, apoptosis, and differentiation during mammalian development [1]. Links between miRNAs and the development of human malignancies have become apparent. Misexpression of cancer-related miRNAs leads to the initiation and progression of cancer by modulating their target oncogenes or tumor suppressor genes [2,3]. We have reported that some important tumor suppressor miRNAs are silenced by epigenetic alterations such as DNA methylation and histone modification in human cancer cells [4,5].

Accumulated evidence has clarified that cancer cells are heterogeneous with a hierarchy of “stemness” in solid cancer tissues [6]. Stem cells have the ability to perpetuate themselves through self-renewal and to generate mature cells of various tissues through differentiation. A subpopulation of cancer cells with distinct stem-like properties is responsible for tumor initiation, invasive growth, and metastasis formation, and these are defined as cancer stem cells (CSCs). As CSCs are considered to be resistant to conventional chemotherapies and radiation therapy, it would be desirable to develop a therapeutic strategy specifically targeting CSCs.

miR-34a was identified as a target of p53 and induces a G1 cell cycle arrest, senescence and apoptosis in response to DNA damage [7,8]. miR-34a is an important tumor suppressor whose expression is epigenetically silenced in various human cancers [9,10]. Enforced expression of miR-34a induces cell cycle arrest, apoptosis, senescence, suppression of epithelial-mesenchymal transition (EMT) and inhibits cell proliferation of CSCs [11]. Here we review about epigenetic silencing of miR-34a in human cancers and a therapeutic strategy targeting CSCs through up-regulating miR-34a expression.

## 2. Biogenesis and Target Genes of miR-34a

**Figure 1:**
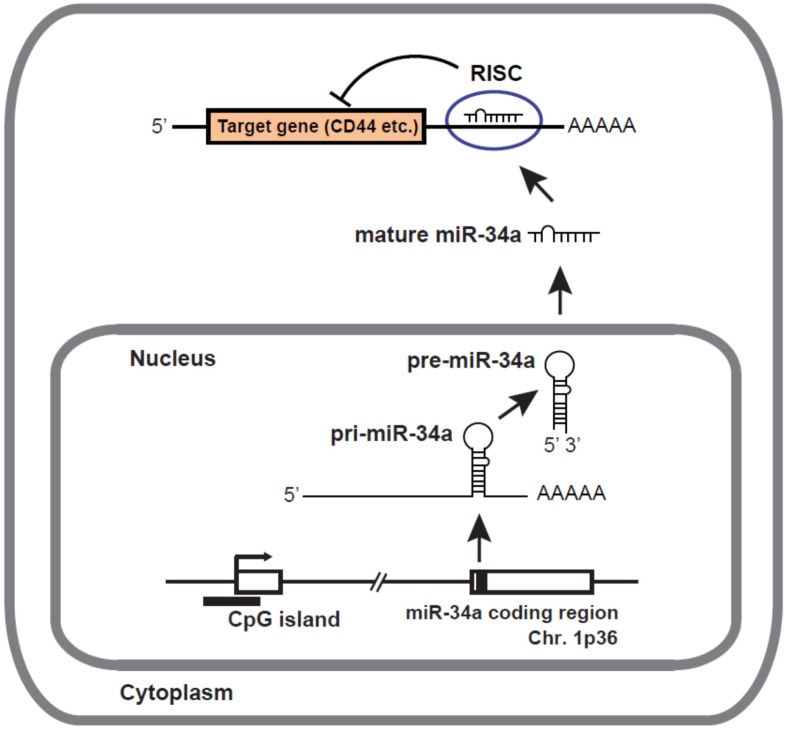
Biogenesis of miR-34a. miR-34a genes are transcribed from TSS by RNA pol II to form pri-miR-34a, which is capped with 7-methylguanosine and polyadenylated (AAAAA). Drosha and its co-factor DGCR8 process pri-miR-34a into pre-miR-34a. Pre-miR-34a is transported into the cytoplasm and subsequently cleaved by Dicer into mature miRNAs. Mature miR-34a is then loaded into RISC, where miR-34a down-regulates specific gene products by translational repression via binding to partially complementary sequences in the 3′UTR of the target mRNAs such as CD44 or by directing mRNA degradation via binding to perfectly complementary sequences.

The miR-34a gene is located at the chromosome 1p36 locus. As shown in Figure 1, the miR-34a gene is transcribed from a transcription start site located in the CpG island by RNA polymerase II (pol II) to form primary transcript (pri-miR-34a). DNA hypermethylation of the CpG island promoter region is one of the most common reasons for silencing of miR-34a [9,10]. Pol II-transcribed pri-miR-34a is capped with 7-methylguanosine and is polyadenylated. The nuclear RNase III enzyme Drosha and its co-factor DGCR8 process pri-miR-34a into precursor miR-34a (pre-miR-34a), which forms an imperfect stem-loop structure. Pre-miR-34a is transported into the cytoplasm by exportin 5 and subsequently cleaved by Dicer into mature miR-34a, which is then loaded into the RNA-induced silencing complex (RISC). The miR-34a/RISC complex down-regulates specific gene products by translational repression via binding to partially complementary sequences in the 3′-untranslated regions (UTRs) of the target mRNAs such as CD44 or by directing mRNA degradation via binding to perfectly complementary sequences.

Identification of target genes of miR-34a is critical to determine its molecular function in cancer cells. A simple biochemical method to isolate mRNAs pulled down with a transfected, biotinylated miRNA was used to identify direct target genes of miR-34a [12]. Transcripts for 982 genes were enriched in the pull-down with miR-34a in K532 and HCT116 cancer cell lines, and most of them were validated as directly regulated by miR-34a. The transcripts pulled down with miR-34a were highly enriched for their roles in growth factor signaling and cell cycle progression. Thus, miR-34a is capable of regulating hundreds of genes associated with growth factor signal transduction and downstream pathways required for cell division.

Moreover, proteome analyses identified early targets of miR-34a that enhance tumor progression including signaling pathways such as TGF-β, WNT and mitogen-activated protein kinase (MAPK) in neuroblastoma [13]. Keller *et al.* [14] combined pulsed SILAC (Stable Isotope Labeling by Amino acids in Cell culture) and microarray analyses to identify alterations in protein and mRNA expression induced by miR-34a. This type of combined approach revealed that miR-34a plays important roles in multiple tumor-suppressive pathways by directly and indirectly suppressing the expression of numerous critical proteins.

## 3. Inactivation of miR-34a in Various Types of Cancers

miRNAs can have large-scale effects through regulation of a variety of target genes during carcinogenesis. Therefore, understanding the regulatory mechanisms controlling miRNA expression is very important. Many miRNAs are expressed in a tissue and tumor specific manner, implying that some miRNAs are under the epigenetic control. Since miR-34a is a direct target of p53, inactivating mutations of p53, increased expression of p53 inhibitors and genomic mutations at the p53-binding site in the miR-34a gene may cause loss of miR-34a expression. In addition, miR-34a resides on the chromosomal locus 1p36, which has been reported to be deleted in human malignancies. Thus, inactivation of the miR-34a gene is a common event during carcinogenesis. Recently, epigenetic inactivation of miR-34a was identified in various types of cancers. Epigenetics is an acquired modification of methylation and/or acetylation of chromatin DNA or histone proteins, which regulates downstream gene expression. Epigenetic alterations can be induced by aging, chronic inflammation, or viral infection, and aberrant DNA methylation and/or histone modification induces inactivation of tumor suppressor genes and play critical roles in the initiation and progression of human cancer [15]. 

We have shown that ~5% of human miRNAs are up-regulated more than three-fold by treatment of T24 bladder cancer cells with the DNA demethylating agent 5-aza-2′-deoxycytidine (5-Aza-CdR) and the histone deacetylase (HDAC) inhibitor 4-phenylbutyric acid (PBA). In particular, miR-127, which is embedded in a CpG island, is remarkably induced by a decrease in DNA methylation levels and an increase in active histone marks around the promoter region of the miR-127 gene. In addition, activation of miR-127 by epigenetic treatment induced down-regulation of its target oncogene BCL6 [4,5]. We have also demonstrated that treatment of gastric cancer cells with 5-Aza-CdR and PBA induces activation of miR-512-5p which is located at Alu repeats on chromosome 19. Activation of miR-512-5p by epigenetic treatment induces suppression of MCL1, resulting in apoptosis of gastric cancer cells [16]. These results indicate that chromatin remodeling by epigenetic therapy can directly activate miRNA expression and re-activation of silenced tumor suppressor miRNAs could be a novel therapeutic approach for human cancers.

A recent study has demonstrated that expression of the tumor suppressor miR-34a is silenced in breast, lung, colon, kidney, bladder and pancreatic cancers as well as melanoma due to aberrant CpG methylation of its promoter region [9]. Re-expression of miR-34a in cancer cell lines induced senescence and cell cycle arrest at least in part by targeting CDK6, indicating that miR-34a represents a tumor suppressor gene which is inactivated by CpG methylation in multiple types of cancer [9]. Epigenetic silencing of miR-34a via DNA hypermethylation of its promoter region is also observed in hematological malignancies such as non-Hodgikin’s lymphoma [10]. The other miR-34 family members, miR-34b and miR-34c, are also reported to be silenced by aberrant CpG island methylation in colorectal cancer [17].

Long, non-coding RNAs (lncRNAs) are important new members of the non-coding RNA family that are greater than 200 nt without protein coding ability. The lncRNA HOX antisense intergenic RNA (HOTAIR) is overexpressed in various malignancies including colon, pancreatic and breast cancer. HOTAIR epigenetically silenced miR-34a expression by recruiting the polycomb repressive complex 2 (PRC2), which results in promotion of epithelial-mesenchymal transition (EMT) in gastric cancer cells [18].

## 4. Biological Effects of miR-34a in CSCs

**Figure 2:**
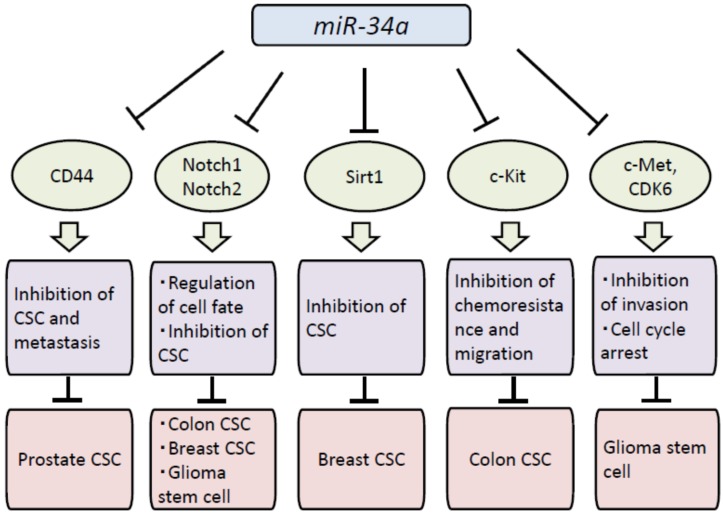
Biological effects of miR-34a in CSCs. The direct targets and biological effects of miR-34a in various CSCs are summarized; CSC; cancer stem cell.

Since miR-34a suppresses many oncogenes and cancer stem cell markers including CD44, CDK4, CDK6, c-Met, Notch-1, Notch-2, SIRT1 and DLL1 as its target genes [11,19,20,21], miR-34a plays important roles in cancer stem cells. The direct targets and biological effects of miR-34a in various CSCs are summarized in Figure 2.

CD44 is one of the important stem cell markers and was validated as a direct and functional target of miR-34a. Enforced expression of miR-34a inhibited prostate cancer stem cells and metastasis by directly repressing CD44, indicating that miR-34a is a key negative regulator of prostate CSCs and could be a novel therapeutic agent against prostate cancers [11]. Notch1 is also an important target of miR-34a and involved in the maintenance and self-renewal of CSCs. miR-34a plays as a cell-fate determinant in early-stage dividing colon CSCs [22] and inhibits breast CSCs and glioma stem cells by regulating Notch1 [23,24]. Gliomas are the most common tumors of central nervous system. The transformation to a glioma stem cell state is involved in aberrant expression of miRNAs including miR-34a. miR-34a suppresses cell proliferation and tumor growth of glioma stem cells by targeting Rictor through its effects on AKT/mTOR pathway and Wnt signaling [25].

Recent studies have revealed that enforced expression of miR-34a suppresses cell proliferation of lung CSCs, colon CSCs, malignant mesothelioma cells and breast CSCs by targeting Arhgap1, c-Kit, c-Met and Sirt1, respectively [26,27,28,29]. These findings indicate that miR-34a is a promising therapeutic agent targeting various CSCs through down-regulation of target oncogenes and stem cell markers.

## 5. miR-34a Is a Promising Therapeutic Agent against Human Cancer 

Chromatin-modifying drugs such as DNA methylation inhibitors and HDAC inhibitors have shown clinical promise for cancer therapy [15,30]. The DNA methylation inhibitor 5-Aza-CdR, which is an analog of cytidine, has been widely studied and was recently approved for the treatment of myelodysplastic syndrome (MDS). The HDAC inhibitor suberoylanilide hydroxamic acid (SAHA) has been approved for patients with cutaneous T-cell lymphoma. Other inhibitors of DNA methylation and HDAC are also in clinical trials.

A promising option for cancer therapy is the use of epigenetic drugs which inhibit tumor growth via several mechanisms, including restoring the expression of epigenetically silenced miR-34a. Inhibitors of DNA methylation and histone deacetylation can work synergistically to suppress the growth of cancer cells. Many epigenetic drugs have shown promising results in clinical trials and recent advances in research suggest a new anticancer effect from this class of drugs. By inducing miR-34a expression, epigenetic therapy not only inhibits the growth of cancers, but may also inhibit the invasiveness and metastatic potential of CSCs. Re-expression of miR-34a by treatment with 5-Aza-CdR and SAHA strongly inhibited cell proliferation, cell cycle progression, self-renewal, EMT and invasion in pancreatic CSCs [31]. Further studies are necessary to develop chromatin-modifying drugs that specifically affect only the CpG island promoter region of miR-34a to reduce the side effects of epigenetic therapy.

Another option for restoring miR-34a expression is replacement therapy using miR-34a mimics. The concept of this therapy is to restore miR-34a expression in cancers to a comparable level to surrounding non-cancer tissues. A recent study has shown that systemic delivery of miR-34a mimics using a neutral lipid emulsion inhibits lung tumors in mice [32]. Mirna Therapeutics (http://www.mirnatherapeutics.com/) is developing MRX34, a mimic of naturally occurring miR-34a encapsulated in liposomal nanoparticle formulation. This has shown preliminary clinical evidence of anti-tumor activity in a Phase 1 clinical trial (NCT01829971). Tissue-specific delivery and cellular uptake of sufficient amounts of synthetic oligonucleotides to achieve sustained target inhibition are very important issues. In particular, biological instability of oligonucleotides in tissues and poor cellular uptake need to be resolved to make miRNA-based therapy successful. Since miRNAs have the ability to simultaneously regulate several cellular pathways, this multi-target property of miRNAs might potentially result in off-target side effects. We have to be careful about these potential side effects for future clinical applications of miRNA-based therapy.

## 6. Conclusions

The tumor suppressor miR-34a plays important roles in the initiation and progression of various types of human malignancies by down-regulating target oncogenes and CSC markers. Restoring of miR-34a expression through epigenetic therapy with inhibitors of DNA methylation and HDAC and/or delivery of miR-34a mimics could be a powerful cancer therapy targeting CSCs. Further studies are needed to develop chromatin-modifying drugs that specifically affect the miR-34a gene and miR-34a mimics that have anti-tumor activity with reduced side effects.
